# Predicting response and survival to first-line treatment with baseline [^18^F]FDG-PET-CT in patients with small-cell lung cancer: an integrated diagnostic approach

**DOI:** 10.1177/17588359251379665

**Published:** 2025-10-01

**Authors:** David Ventura, Philipp Schindler, Peter Kies, Annalen Bleckmann, Michael Mohr, Georg Lenz, Michael Schäfers, Wolfgang Roll, Georg Evers

**Affiliations:** Department of Nuclear Medicine, University Hospital Münster, Albert-Schweitzer-Campus 1, Münster, NRW 48149, Germany; West German Cancer Center (WTZ), Germany; European Institute for Molecular Imaging (EIMI), University of Münster, Münster, Germany; West German Cancer Center (WTZ), Germany; Clinic for Radiology, University and University Hospital Münster, Münster, NRW, Germany; Department of Nuclear Medicine, University Hospital Münster, Münster, NRW, Germany; West German Cancer Center (WTZ), Germany; West German Cancer Center (WTZ), Germany; Department of Medicine A-Hematology, Oncology, Hemostaseology and Pneumology, University Hospital Münster, Münster, NRW, Germany; West German Cancer Center (WTZ), Germany; Department of Medicine A-Hematology, Oncology, Hemostaseology and Pneumology, University Hospital Münster, Münster, NRW, Germany; West German Cancer Center (WTZ), Germany; Department of Medicine A-Hematology, Oncology, Hemostaseology and Pneumology, University Hospital Münster, Münster, NRW, Germany; Department of Nuclear Medicine, University Hospital Münster, Münster, NRW, Germany; West German Cancer Center (WTZ), Germany; European Institute for Molecular Imaging (EIMI), University of Münster, Münster, Germany; Department of Nuclear Medicine, University Hospital Münster, Münster, NRW, Germany; West German Cancer Center (WTZ), Germany; West German Cancer Center (WTZ), Germany; Department of Medicine A-Hematology, Oncology, Hemostaseology and Pneumology, University Hospital Münster, Münster, NRW, Germany

**Keywords:** FDG-PET, integrated model, PFS/OS prediction, radiomics, SCLC

## Abstract

**Background:**

Small-cell lung cancer (SCLC) is a highly malignant disease with a propensity for early progression and high mortality. The prognostic value of treatment response and survival has been verified for solely established imaging, clinical, and biochemical markers. There is a lack of evidence for the combination of those parameters with machine learning and integrated models, particularly in the context of molecular imaging.

**Objectives:**

The aim of this study was to predict early disease progression and survival using CT-based radiomic features (RF), integrating [^18^F]FDG-PET-CT and clinical parameters.

**Design:**

This retrospective pilot study included 62 patients with non-metastatic and metastatic SCLC who underwent stage-based primary treatment following baseline [^18^F]FDG-PET-CT. The development of a machine learning approach, incorporating clinical and molecular imaging parameters, enables the creation of a model capable of predicting treatment response and survival.

**Methods:**

A radiomics signature was generated based on the first-line treatment response by RECIST 1.1 criteria. The RF was integrated using binary logistic regression analysis with the PET parameter metabolic tumor volume (MTV) of the primary tumor and initial disease stage. The integrated model with the highest AUC for predicting early disease progression was evaluated for predicting progression-free survival (PFS) and overall survival (OS) in both non-metastatic and metastatic patients.

**Results:**

A single CT-based RF demonstrated predictive capacity (AUC = 0.81). Integration of the MTV and disease stage enhanced the predictive capacity (AUC = 0.9). A Youden index-based threshold of <0.62 was identified as a significant predictor of prolonged PFS: non-metastatic disease with a median PFS of 25 versus 4 months (HR = 0.072; *p* = 0.002); metastatic disease with a median PFS of 9 versus 5 months (HR 0.219; *p* = 0.004). The integrated model also predicted OS in metastatic disease with a median OS of 15 versus 8 months (HR 0.381; *p* = 0.013).

**Conclusion:**

A multiparametric approach based on a Radiomics model may potentially be capable of identifying patients at risk for early disease progression, PFS, and OS in non-metastatic and metastatic SCLC.

## Introduction

Lung cancer is one of the most common malignant tumor entities, with small cell lung cancer (SCLC) accounting for approximately 15% of primary lung tumors.^1^ SCLC is a poorly differentiated, highly aggressive malignancy that arises from the neuroendocrine cells of the lung parenchyma and is common in heavy smokers.^2^ Due to these pathognomonic features, SCLC often presents with high tumor load and advanced stages at initial diagnosis. However, today, SCLC is recognized as a biologically heterogeneous diagnostic category in which different molecular genetic characteristics may be associated with potentially different treatment options, thus influencing the biology and therapeutic susceptibilities of SCLC.^3^ In addition, intertumoral heterogeneity highlights the complexity of SCLC and represents a potential obstacle to treatment strategies for recurrent SCLC.^4^

In the vast majority of cases, SCLC is diagnosed at a locally advanced or metastatic stage at the time of initial diagnosis.^4^ Patients with locally advanced disease are usually treated with combined radio-chemotherapy with curative intent, whereas patients with primarily distant metastatic disease are treated with combined chemo-immunotherapy with palliative intent. The prognosis for both metastatic and locally advanced disease is generally poor, with the majority of patients dying from disease progression or recurrence. The rapid development of resistance to therapy and the lack of biomarkers to effectively select patients with SCLC remains a constant challenge.^4^

Tumor stage at initial diagnosis can be used as a key prognostic parameter, as the proportion of patients with long-term survival in locally advanced disease is significantly higher than in patients with primarily metastatic disease, most of whom die of disease progression within 2 years.^5^ However, the ability to accurately predict early disease progression based on the initial stage of the disease is limited.^6^ Apart from biological tumor characteristics, tumor imaging parameters have been considered for the characterization of tumor heterogeneity as well as for prediction of disease progression and survival.^7^ It is therefore proposed that the integration of available routine biomarkers and parameters in machine learning algorithms may assist in the stratification of patients undergoing treatment.

[^18^F]fluorodeoxyglucose positron-emission-tomography computed-tomography (PET-CT) is an important part of the initial staging of SCLC. It is used to select the optimal treatment regime based on the stage of the disease.^8,9^ Studies have shown that standard semiquantitative PET parameters as maximum standardized uptake value (SUVmax), metabolic tumor volume (MTV) have a prognostic impact in SCLC.^10,11^ Nevertheless, these conventional parameters, including CT-based assessments of size and density, are inadequate for providing more detailed insights into tumor heterogeneity.^7^ Therefore, parameters and biomarkers that go beyond standard assessments are needed to characterize heterogeneous disease patterns.^12,13^

Advanced quantitative imaging parameters can be extracted from CT and PET images in a high-throughput manner, generating a large amount of data.^14^ The extraction of encrypted image data into mineable numeric data requires artificial intelligence (AI) and machine learning (ML) analysis.^12^ Predicting treatment response with a PET-based Radiomics model has already shown promising results in NSCLC.^7,15,16^ In SCLC, some studies have shown promising results for treatment prediction using morphological imaging with CT in a Radiomics model.^17–19^ However, to date, there have been no reported studies investigating the role of a Radiomics model including PET- and CT-based parameters in SCLC.

Therefore, the aim of the study was to determine whether a Radiomic model incorporating PET, CT-, and clinical data can predict early disease progression and survival in patients with SCLC.

## Material and methods

### Study design

This is a retrospective, single-center observational study in a tertiary care academic medical center. All patients received first-line treatment according to pivotal study protocols. In patients with non-metastatic disease a platinum-etoposide and radiation-based treatment protocol was administered.^20^ The majority of these patients (39/62, 62.9%) also underwent prophylactic cerebral radiation following radiochemotherapy. In metastatic disease patients received either aforementioned chemotherapy protocol (before 2018) or an ICI-based immunochemotherapy protocol with Atezolizumab.^20,21^

This study was approved by the local ethics committee (Number 2022-391-f-S, Ethics Commission of the Medical Association Westphalia-Lippe and the University of Muenster). This study was conducted in accordance with the ethical standards of the Declaration of Helsinki of 1964 and its subsequent amendments.

### Patient selection

Patients treated between 2013 and 2022 were included based on the following criteria: (a) histologically confirmed SCLC; (b) baseline PET with follow-up clinical and imaging data for response evaluation; (c) approval of the interdisciplinary lung tumor board for guideline-based therapy protocols; (d) completed first-line therapy regime; (e) date of death or last follow-up from the cancer registry; (f) age > 18 years.

### Baseline PET-CT

All patients underwent baseline PET-CT prior to treatment initiation based on literature recommendations.^7,22^ Image acquisition was performed using a Siemens Biograph mCT 128 system (Siemens Healthcare, Erlangen, Germany). 3 MBq/kg body weight [^18^F]FDG was administered intravenously after at least 6 h of fasting (blood glucose < 6mM). PET images with additional low-dose CT-scan for attenuation correction from skull base to proximal femur were acquired 60 min after tracer injection. An additional contrast-enhanced CT scan of the chest was obtained in the venous phase 45 s after intravenous injection of the contrast agent (Ultravist 300, Bayer Vital GmbH, Germany).

### CT-based response and survival evaluation

All imaging, patient, and procedural data were obtained retrospectively from the electronic medical record and the image archiving and communication system. Clinical data and therapy validations were retrieved from the electronic health records. First and second follow-up imaging were performed after treatment initiation to assess treatment response using a contrast-enhanced CT scan. The first follow-up was performed at a median interval of 2.1 months (95% CI: 1.6–2.3 months) as an interim assessment, and the second follow-up was performed at a median interval of 4.6 months (95% CI: 4.3–4.9 months) after the completion of the primary treatment protocol. At these two time points, a standardized objective assessment of treatment response was performed by an experienced radiologist. For this purpose, serial segmentation of the primary tumor was performed on contrast-enhanced CT chest scans using the mint Lesion^™^ software package version 3.9.5 (Mint Medical GmbH, Heidelberg, Germany). Evaluation was based on the current “Response Evaluation Criteria in Solid Tumors” (RECIST 1.1)^23^: complete response (CR) was defined as complete reduction of the primary tumor; partial response (PR) as at least 30% reduction and progressive disease (PD) as at least 20% increase in the longest diameter with respect to the sum of baseline diameters. If the criteria for PR or PD were not met, stable disease (SD) was defined in terms of the smallest sum diameter. Disease control rate (DCR) was defined as either CR, PR or SD. Patients with no follow-up information available after a certain time were classified as “lost to follow-up.” The final Radiomic analysis was based on treatment response (progression vs non-progression) to therapy at the second follow-up after the completion of the primary treatment protocol. A further imaging-based comprehensive follow-up was conducted, with the objective of evaluating PFS in patients subjected to PD, taking into account the entire disease.

### Image segmentation and standard parameter extraction of baseline PET-CT

Semi-automatic segmentation through volumetric region of interest (ROI) of the primary tumor was performed by an experienced nuclear medicine and radiology senior physician using mint Lesion™. Readers selected the primary tumor in both PET and CT images, defined as:

- PET: According to the “Positron Emission Response Criteria In Solid Tumors” (PERCIST 1.1), a threshold of 1.5 × mean liver “standardized uptake value” (SUV_MEAN_) + 2 standard deviations was used to define the [^18^F]FDG-positive “volume of interest” (VOI) of the primary tumor.^24^ For the primary tumor side, standard PET parameters were obtained for each patient: SUV_PEAK_ (1 cm^3^ VOI), SUV_MAX_, SUV_MEAN_, SUV_MIN_, metabolic tumor volume (MTV of 40% adapted SUV-body weight), and total lesion glycolysis (TLG) defined as SUV_MEAN_ × MTV.^25^
- CT: The ROI of the primary tumor region was delineated in the late arterial phase of a contrast-enhanced CT scan in accordance with the established RECIST 1.1 criteria.^23^

### Radiomic feature selection and analysis

Following the guidelines of the Image Biomarker Standardization Initiative (IBSI), 57 radiomic features (RFs) including morphology, intensity-based, histogram-based (first-order texture), and gray-level co-occurrence matrix (GLCM, second-order texture) features were extracted for the primary lung tumor in both PET and CT separately.^26^ All extracted radiomics features were standardized using *z*-score normalization to ensure comparability. For the selected RF, the concordance correlation coefficient was calculated to assess the reliability of the features between two readers. Thus, RFs with a coefficient between 0.8 and 1 were classified as “excellent” and included in further analysis.^27^

For further feature reduction, the dataset was split into training and validation subsets with a 70:30 ratio for a fixed hold-out set of patients to provide an unbiased estimate for further analysis. The trainings-set population of 70% of the included patients meet the recommended TRIPOD+AI (Transparent Reporting of a multivariable prediction model for Individual Prognosis Or Diagnosis) statement and PROBAST+AI (The Prediction model Risk Of Bias ASsessment Tool) framework for the use of ML models.^28,29^ Using the reliable RF, a radiomics signature was developed to predict treatment response in the training set. Feature reduction was performed using the Least Absolute Shrinkage and Selection Operator (LASSO) algorithm on the training dataset only. A 10-fold cross-validation within the training dataset was used to optimize the LASSO model. The regularization parameter (λ) was determined based on the cross-validated mean squared error. Finally, significant RFs from the training dataset were further tested on the validation dataset using a logistic regression model to select features that allowed for response differentiation. The performance of the model was assessed by constructing a receiver operating characteristic (ROC) curve. RF selection and dimension reduction were performed by using an open-source software package (R/R Studio, version 4.0.5; R Foundation, Austria).

### Statistical analysis

Demographic and clinical data are presented as totals, percentages, medians, ranges, and 95% confidence intervals (95% CI). ROC analysis, with area under the curve (AUC) and Youden J for calculating the optimal threshold, was used to determine the value of different individual parameters. Parameters for integration were selected by univariate analysis. A multivariable logistic regression model based on a combination of significant clinical, PET-based, and radiomic parameters for calculating the probability of the given outcome at second follow-up (DCR vs PD) was employed. A ROC analysis with AUC was used to calculate the optimal threshold (1.000-fold bootstrap-validated) with Youden J for predicting response at second follow-up (DCR vs PD) for combined parameters, The Kaplan–Meier analysis with log-rank test (Mantel-Cox) was used to predict PFS and OS with the significant integrated parameter for separated groups (non-metastatic and metastatic). Cox-Regression was used to calculate hazard ratios (HR). *p*-Values < 0.05 were set and considered statistically significant. Statistical and survival analyses were performed using SPSS Statistics version 26 (SPSS Inc., Chicago, IL, USA).

## Results

### Patients’ characteristics

Between December 2013 and October 2022, a total of 62 patients (33 males [53.2%], 29 females [46.8%]) with a median age of 64.5 years (range: 34–84 years) met our previously defined inclusion criteria. All patients were treatment naive. A total of 27 patients (43.5%) were classified as having non-metastatic disease according to the TNM UICC 8 classification, while 35 patients (56.5%) were classified as having metastatic disease.^30^ Detailed patients’ characteristics for the separated groups are demonstrated in Table 1.

**Table 1.** Baseline patients’ characteristics.

Characteristic	*n*	%	Median	Range
Patients	62			
Male	33	53.2		
Female	29	46.8		
Age (years)			64.5	34–84
Smoking status (pack years)			40	15–150
Current	38	61.3		
Former	24	38.7		
ECOG				
ECOG 0	7	11.3		
ECOG 1	36	58.1		
ECOG 2	16	25.8		
ECOG 3	3	4.8		
Disease manifestation				
Thoracic lymph node metastases	54	87.1		
Organ metastases	38	61.3		
Bone	17	44.7		
Brain	16	42.1		
Liver	14	36.8		
Adrenal	10	26.3		
Pulmonal	8	21.1		
UICC 8 stage^30^				
IIA^a^	1	1.6		
IIB	5	8.1		
IIIA	11	17.7		
IIIB	7	11.3		
IV	38	61.3		
Therapy protocols				
	Non-metastatic disease (*n* = 27)		Metastatic disease (*n* = 38)	
First-line treatment	*n*	%	n	%
Carboplatin/Etoposide	6	22.2	6	17.1
Cisplatin/Etoposide	21	77.8	8	22.9
Carboplatin/Etoposide/Atezolizumab	N/A	N/A	21	60.0
Thoracal radiation	27	100	N/A	N/A
PCI	26	96.3	N/A	N/A
Second-line treatment	8	29.6	13	37.1

a This patient underwent primary radio-chemotherapy due to severe obstructive pulmonary disease and was therefore not feasible for surgery.

ECOG, European Cooperative Oncology Group; N/A, not applicable; PCI, Prophylactical Cranial Irradiation.

### Response to treatment at second follow-up

A second follow-up with contrast-enhanced CT imaging was performed to assess response on first-line therapy according to RECIST 1.1 criteria of the primary tumor.^23^ In the group of non-metastatic disease (*n* = 27/62, 43.5%), a total of 6 (22.2%), 12 (44.4%), and 2 (7.4%) patients had CR, PR, and SD (DCR = 74.1%), while 7 patients (25.9%) suffered from PD. In the group of metastatic disease (n = 35/62, 56.5%), a total of 4 (11.4%), 11 (31.4%), and 5 (14.3%) had CR, PR, and SD (DCR = 57.1%), while 15 patients (42.9%) suffered from PD, respectively.

**Figure 1.**
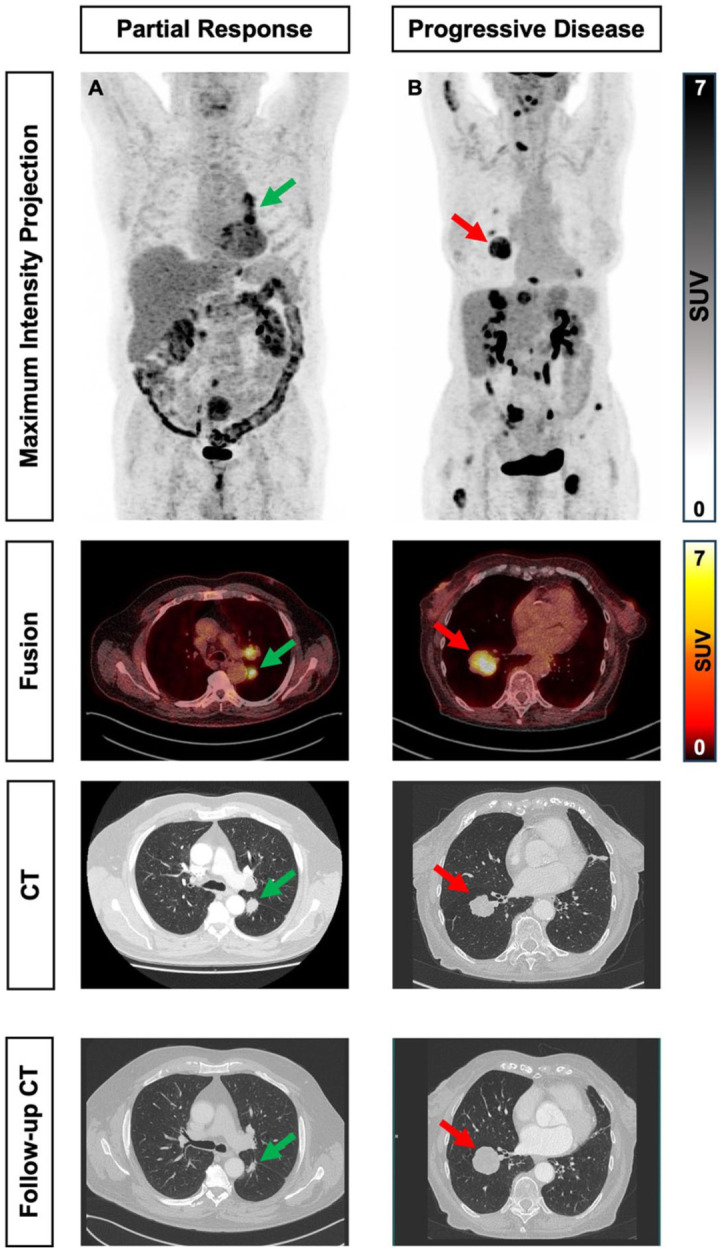
Response assessment to therapy. A 67-year-old male received carboplatin, etoposide, and atezolizumab and revealed PR according to RECIST 1.1 criteria at the second follow-up (a). A 54-year-old female received the same aforementioned treatment protocol and exhibited PD according to RECIST 1.1 criteria at the second follow-up (b).

Figure 1 presents a graphical illustration of the response to therapy of one patient from each group: DCR with PR (Figure 1(a)) and PD (Figure 1(b)) with metastatic disease.

### Prediction of early disease progression with integrated diagnostics in the test cohort

Following the radiomics analysis of the data, one radiomic CT feature, namely, “Firstorder.Intensity.Quartile_Coefficient_Dispersion” (CT_FIQCD) representing tumor imaging micro-heterogeneity due to the composition of grey scales, was identified as having predictive value to early progression at the second follow-up imaging in the test cohort (*n* = 18; 6 patients with non-metastatic and 12 patients with metastatic disease). In contrast, no radiomic PET feature was found to be predictive. CT_FIQCD demonstrated the ability to effectively discriminate between patients with CR/PR/SD versus PD. Patients with a higher score (threshold: >0.5) had a higher degree of imaging tumor micro-heterogeneity and were more likely to progress on first-line treatment. For prediction of early disease progression at second follow-up imaging, the AUC-ROC of CT_FIQCD was 0.81 (sensitivity: 0.67, specificity: 1; Figure 2(a), red line).

**Figure 2.**
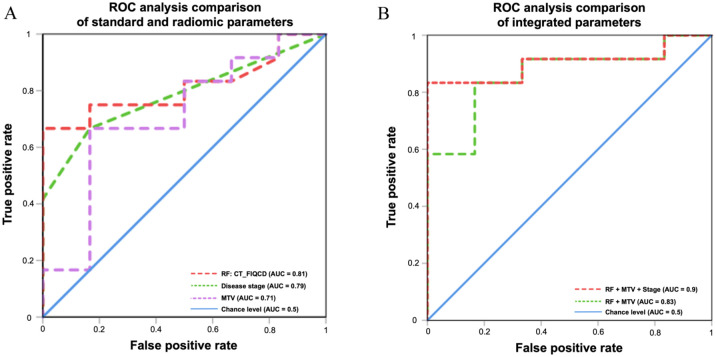
ROC analysis of standard, radiomic, and integrated parameters. ROC analysis of selected parameters that were significant (*p* < 0.05) in univariate analysis. The radiomic feature CT_FIQCD, the disease stage, and the metabolic tumor volume revealed AUC values ranging from 0.71 to 0.81 (a). The application of binary logistic regression to the combined parameters yielded the highest AUC value of 0.9 (b).

The use of isolated PET-based and clinical standard parameters showed a lower ability than CT_FIQCD to predict early disease progression. For example, MTV (median: 18.6, range: 7.8–246.6) showed an AUC of 0.71 in the ROC analysis (Figure 2(a), purple line). The predictive value of multi-parametric approaches was enhanced by the use of binary logistic regression, which facilitated the calculation of probabilities. The integration of the robust standard PET parameter MTV to CT_FIQCD, revealed an AUC of 0.86 (sensitivity: 0.83, specificity: 0.88, threshold: 0.66, Figure 2(b), green line) through ROC analysis. Several clinical parameters (e.g., ECOG, smoking status, or disease stage at diagnosis) were subjected to univariate analysis in order to determine their influence on treatment response at the second follow-up. Here, the only parameter that could discriminate between patients with early progression at the time of the second follow-up and patients with no evidence of progression (DCR) was the initial tumor stage (non-metastatic vs metastatic disease; *p* = 0.03). For this parameter, the ROC analysis showed an AUC of 0.79 (sensitivity: 0.83, specificity: 0.76, threshold: 0.61; Figure 2(a), green line) for determining PD versus CR / PR / SD. To test whether the previously identified imaging parameters (MTV, CT_FIQCD) in combination with initial tumor stage (metastatic vs non-metastatic disease) could further improve the predictive value. The binary logistic regression and final ROC analysis resulted in an AUC of 0.9 (sensitivity: 0.83, specificity: 1, threshold: 0.59, Figure 2(b), red line).

### Prediction of PFS and OS of the entire cohort using the integrated model for non-metastatic and metastatic disease

The radiomic model and the standard PET parameters were based on data obtained from the primary tumor for both groups. The final ROC analysis of the total cohort revealed an AUC of 0.902 for the parameters of the aforementioned integrated model with a threshold of 0.62. Accordingly, patients with a threshold over 0.62 (Youden J: 0.41, sensitivity 83%, specificity 100%) are associated with a higher risk of disease progression (at second follow-up imaging) in both patients with non-metastatic and metastatic disease. The final threshold was calculated with the equation as follows:

$$\begin{array}{l} logit\left(threshold\right)=\;-3.47+5.92★\;\left(CT_{FIQCD}\right)+\\ 0.027★\;\left(MTV\;in\;cm^{3}\right)+1.84★\;\left(Stage\right)\\ threshold=\;\frac{1}{1+\exp[-\left(\begin{array}{l} -3.47+(5.92★CT_{FIQCD})+\\ \left(0.027★MTV)+(1.84★Stage\right) \end{array}\right)]} \end{array}$$

**Figure 3.**
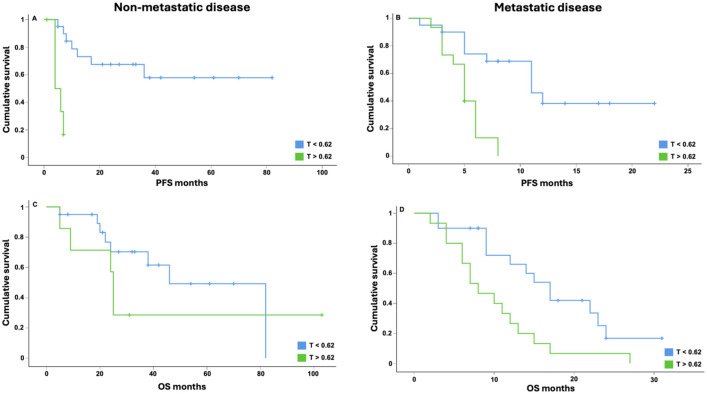
PFS and OS analysis in non-metastatic and metastatic disease. The Kaplan–Meier analysis and the Cox regression model were employed to assess the integrated parameter, with a threshold of 0.62 applied to the entire cohort, categorized into non-metastatic and metastatic disease groups. Patients exhibiting a threshold below 0.62 of the integrated parameters have been shown to demonstrate a significantly prolonged PFS in both groups (a and b). For OS analysis the threshold below 0.62 was not significant in the non-metastatic group (c), but in the metastatic group (d).

It is noteworthy that the integrated model exhibited a significant increase in the PFS of patients with a threshold below 0.62, when analyzing the total PFS for the separated groups (metastatic vs non-metastatic). For non-metastatic disease (*n* = 27) the median PFS was found to be 25 vs 4 months (HR, 0.072; 95% CI, 0.014–0.384, *p* = 0.002; Figure 3(a)). For metastatic disease (*n* = 35), the median PFS was found to be 9 vs 5 months (HR, 0.219; 95% CI, 0.077 – 0.623, *P* = 0.004; Figure 3(b)). In terms of OS analysis for the separated groups (metastatic vs non-metastatic), the integrated model revealed a significant longer OS for patients with a threshold below 0.62, but only for the metastatic group. For non-metastatic disease (*n* = 27), the median OS was found to be 29 vs 25 months (HR, 0.564; 95% CI, 0.338–0.919, *p* = 0.325); Figure 3(c)). For metastatic disease (*n* = 35), the median OS was found to be 15 vs 8 months (HR, 0.381; 95% CI, 0.128–0.816; *p* = 0.013; Figure 3(d)).

## Discussion

Despite the addition of checkpoint inhibitors to conventional chemotherapy in metastatic and non-metastatic disease, SCLC patients are still characterized by poor outcomes, with the majority of patients experiencing disease progression within a few months of initiating first-line therapy.^31–33^ Even in patients with initially non-metastatic disease, the majority of patients die of disease recurrence after initial curative radio-chemotherapy.^34–37^ However, response to therapy and outcome vary substantially from patient to patient, even within the same disease stages.^6^ Established tumor staging systems (e.g., American Joint Committee on Cancer (AJCC)) are widely accepted and used as a tool to predict survival.^30^ However, the utility of stage-based risk stratification as a prognostic tool may be limited due to considerable tumor heterogeneity. This is due to the fact that the definition of the stadium is based on numerical, morphological, and metabolic standard imaging parameters, without consideration of tumor heterogeneity.^38^ The integration of high-throughput clinical and imaging parameters is a promising avenue for improving of currently hampered risk stratification.^39^

The objective of the present study was to determine whether a radiomics model incorporating PET, CT, and clinical parameters could identify different risk groups of patients with SCLC. The integration of reliable, uniform parameters has resulted in an improvement in the model’s ability to anticipate early disease progression, as well as PFS and OS for the separate groups (non-metastatic and metastatic). The results of our study suggest that the integration of clinical data with advanced imaging features (CT and PET/CT) may prove to be a tool for risk stratification in SCLC patients independent of initial tumor stage (non-metastatic vs metastatic).

### CT-based radiomics model

Radiomics is an emerging discipline that uses ML algorithms to derive quantitative, high-throughput parameters from standard imaging techniques. This approach has the potential to optimize treatment stratification in a manner consistent with the principles of personalized medicine, particularly in the context of lung cancer.^38^ In recent years, several studies have demonstrated the potential of CT-based Radiomics models in lung cancer, focusing mainly on NSCLC with promising results.^15^ In SCLC, the most comprehensive studies have included a heterogeneous cohort of patients, investigating the primary tumor site and including all disease stages.^17,18^ However, this approach is based on the premise that a Radiomic model can be used to characterize the heterogeneity and shape of the primary tumor site, which is likely to differ between patients with localized and metastatic disease.^40^ The results of our investigation showed that a CT-based RF may be an effective prognostic indicator for both predicting response to first-line treatment and survival in a heterogeneous group of patients. In a similar patient cohort and investigation to our study, Gkika et al. (2020) reported that no predictive RF was identified.^41^ Zheng et al. integrated a number of significant clinical parameters into the Radiomics model in order to optimize the integrated nomogram for PFS prediction.^17^ To the best of our knowledge, there has been no investigation of the integration of additional standard PET-parameters into a CT-based Radiomics model, although PET is also widely used in the initial staging of SCLC patients.

### Integration of clinical- and standard PET parameters

In recent years, several studies have explored the extension of nomograms to disease prognostication using standard parameters and biomarkers.^42^ Su et al. demonstrated that a nomogram including significant clinical parameters identified by Cox regression analysis (e.g., treatment modality), outperformed the TNM classification in predicting PFS in patients with SCLC.^18^ In our study, the initial disease stage (metastatic vs non-metastatic) was identified as a prognostic factor in a Cox regression and ROC analysis and was therefore integrated into the radiomics model.

Single standard PET parameters, such as SUVmax and MTV reflecting the metabolic activity and glucose consumption of a tumor, have been shown to be predictive parameters in SCLC patients.^10,43^ Consequently, the incorporation of standard PET parameters alone into clinical routines for the management of individual patients could be contemplated. This is particularly relevant for patients who are suspected to have a higher risk of progression and mortality based on the PET metrics. Moreover, a recent meta-analysis conducted by Christensen et al. demonstrated that the PET parameters SUVmax and MTV are also prognostic indicators for survival.^43^ MTV was identified as the PET parameter with the highest predictive value for response to the stage-based primary treatment protocol and was therefore included in the final model. The incorporation of supplementary PET parameters enhanced the accuracy of outcome prediction, as evidenced by a preceding publication that demonstrated the predictive value of radiomic PET-based parameters in metastatic NSCLC patients.^7^ In the analysis of our SCLC cohort, the validation of the integrated across the entire cohort, divided into metastatic and non-metastatic groups, was found to delineate disparate risk groups with respect to PFS and OS. Therefore, patients at a higher risk of progression and mortality, as calculated by our threshold, may benefit from early therapeutic intensification and advanced monitoring.

The value of prognostic biomarkers like neutrophil-to-lymphocyte ratio (NLR) and circulating tumor cells (CTC) has been identified as factors that influence the OS for SCLC patients. As posited by Liu et al. (2017), patients exhibiting elevated NLR levels demonstrated a twofold elevated risk (HR: 2.09) of mortality. Hiltermann et al. (2012) observed a threefold elevated risk (HR: 3.4) for the presence of detectable CTC prior to treatment initiation.^[Bibr bibr44-17588359251379665] 45^ The integrated model demonstrated comparable HRs for OS risk stratification. The collection of NLR and CTC has not been systematically conducted in the present cohort. The incorporation of prognostic biomarkers has the potential to enhance the efficacy of multiparametric integrated models. It is recommended that the integration of PET parameters and clinical markers be further investigated in the context of larger prospective and multicenter datasets. Furthermore, the integrated model will be validated using external multicentric data prospectively. In view of the expanding molecular genetic knowledge of SCLC heterogeneity, subsequent analyses of treatment response and survival should incorporate tumor biology parameters in conjunction with prognostic biomarkers, as well as clinical and imaging parameters.

### Limitations

This study is limited by its retrospective approach and small patient cohort, which was further divided into a training and a validation set. Consequently, a survival analysis of the test cohort’s subgroups (*n* = 18) was not viable due to the limited patient numbers. Nevertheless, as a pilot study, the aforementioned study design met the best-practice recommendations for early-phase retrospective modeling (TRIPOD statement and PROBAST framework). The collated data provide a rationale for a prospective multicenter approach to validate the results obtained.

For our radiomics analysis, we included patients with both metastatic and non-metastatic tumor stages, which resulted in a heterogeneous patient population. Furthermore, patients with metastatic disease received a different first-line treatment before March 2020 (chemotherapy only) due to the approval status of the immunotherapy. It should also be noted that the examination times of the follow-up imaging, which formed the basis of our analysis, were understandably not uniform due to the real-world setting.

## Conclusion

This is the first study to investigate the role of a Radiomics model incorporating both PET- and CT-based Radiomic parameters in SCLC. Our analysis suggests that an integrated model based on ML approaches, incorporating parameters from PET and CT, may allow to identify different risk groups in patients with non-metastatic and metastatic SCLC.

## Declarations

**Ethics approval and consent to participate:** This study was performed in line with the principles of the Declaration of Helsinki. Approval was granted by the Ethics Committee of the University of Münster (approval number 2022-391-f-S). Written informed consent was obtained from all patients.

**Consent for publication:** All participants gave informed consent.

**Availability of data and materials:** Upon reasonable request, the data generated and/or analyzed are available from the corresponding author.
